# Digitale Innovationen in der Impfkommunikation

**DOI:** 10.1007/s00103-025-04019-3

**Published:** 2025-02-13

**Authors:** Robert Böhm, Rian Gross, Sabrina Forst, Julia Reiter, Cornelia Betsch

**Affiliations:** 1 Fakultät für Psychologie, Universität Wien, Universitätsstraße 7, 1010 Wien, Österreich; 2 Department für Psychologie und Center for Social Data Science, Universität Kopenhagen, Kopenhagen, Dänemark; 3 Gesundheitskommunikation, Institute for Planetary Health Behaviour, Universität Erfurt, Erfurt, Deutschland; 4 Gesundheitskommunikation, Implementationsforschung, Bernhard-Nocht-Institut für Tropenmedizin, Hamburg, Deutschland

**Keywords:** Impfmüdigkeit, Soziale Medien, Smartphone-Apps, Chatbots, Virtuelle Realität, Vaccine hesitancy, Social media, Smartphone apps, Chatbots, Virtual reality

## Abstract

Trotz des großen Erfolges von Impfungen stellt die zunehmende Impfmüdigkeit eine Bedrohung für die öffentliche Gesundheit dar. Deshalb ist eine effektive Impfkommunikation wichtig. Sowohl personalisierte und bedürfnisgerechte Gespräche zwischen Ärzt*innen und Patient*innen als auch großangelegte standardisierte Impfkampagnen über klassische Medien zählen zu den wichtigsten Werkzeugen, um mögliche Unsicherheiten und Sorgen von Patient*innen zu entkräften, Fehlinformationen zu korrigieren und positive Argumente für den Nutzen von Impfungen zu vermitteln. In diesem Beitrag werden digitale Innovationen für die Impfkommunikation vorgestellt, die Vorteile beider Kommunikationsformen kombinieren und damit wichtige Bausteine für die Impfkommunikation in einer zunehmend digitalisierten Gesellschaft werden können. Wir betrachten 4 digitale Ansätze: soziale Medien, Smartphone-Apps, Chatbots und immersive Technologien (insbesondere virtuelle Realität). Wir beschreiben die Eigenschaften dieser Medien und Technologien, mögliche Zielgruppen für ihre Anwendung, ausgewählte wissenschaftliche Evidenz zu ihrer Effektivität in der Impfkommunikation sowie Forschungslücken und -potenziale. Abschließend machen wir Vorschläge für mögliche Anwendungs- und Einsatzbereiche dieser Ansätze in der Impfkommunikation. Auch wenn die Forschung zu digitalen Innovationen in der Impfkommunikation noch am Anfang steht, sehen wir große Potenziale, diese Ansätze als ergänzende Maßnahmen in umfassende Impfkommunikationsstrategien zu integrieren, um die Effektivität zukünftiger Impfkampagnen zu steigern und die globale Gesundheit nachhaltig zu fördern.

## Abstract (en)

Despite the significant success of vaccinations, increasing vaccine hesitancy poses a threat to public health, making effective vaccination communication essential. Both personalized, needs-based conversations between healthcare providers and patients and large-scale, standardized vaccination campaigns through traditional media are important tools for addressing patients’ concerns, correcting misinformation, and conveying positive arguments for the benefits of vaccination. This article describes innovative digital solutions for vaccination communication that combine the strengths of these two approaches, potentially becoming vital components of vaccination communication in an increasingly digital society. We focus on four digital solutions: social media, smartphone apps, chatbots, and immersive technologies (particularly virtual reality). We describe the features of these media and technologies, potential target groups for their application, selected scientific evidence regarding their effectiveness in vaccination communication, and potential research gaps and opportunities. Finally, we offer suggestions for possible application areas of these digital solutions in vaccination communication. While research on the use of innovative digital solutions in vaccination communication is still in its early stages, we see great potential for integrating these technologies as complementary measures into comprehensive vaccination communication strategies to enhance the effectiveness of future vaccination campaigns and promote global health.

## Hintergrund

Trotz der großen Erfolge von Impfungen [1] haben Impfprogramme weltweit mit einer zunehmenden Impfmüdigkeit der Bevölkerung zu kämpfen, die durch verzögerte oder abgelehnte Impfungen zu einem Rückgang der globalen Impfraten führt und die öffentliche Gesundheit gefährdet [2, 3]. Aufgrund der vielfältigen Ursachen für Impfmüdigkeit [4, 5] ist eine effektive, personalisierte Impfkommunikation von zentraler Bedeutung, um möglichen Unsicherheiten und Sorgen zu begegnen, Fehlinformationen zu korrigieren und positive Argumente für den Nutzen von Impfungen zu vermitteln [6].

Das persönliche Gespräch zwischen Ärzt*in und Patient*in ist einer der wichtigsten Kanäle für die Impfkommunikation. Ärzt*innen, insbesondere Hausärzt*innen, genießen in der Regel hohes Vertrauen [7] und können individuelle Informationslücken oder Bedenken im Dialog gezielt adressieren (siehe [8] für einen Überblick). Tatsächlich erleben viele Ärzt*innen eine umfassende und effektive Impfkommunikation in Anbetracht zunehmender Unsicherheiten und Zeitmangels als herausfordernd [9]. Deshalb, und auch um eine breitere Zielgruppe zu erreichen, werden seit Jahrzehnten Impfkampagnen über klassische Medien wie Fernsehen, Radio und Printmedien durchgeführt. Plakate und Informationsbroschüren in Praxen von Ärzt*innen, in Apotheken und im öffentlichen Raum spielen ebenfalls eine wichtige Rolle. Diese traditionellen Kommunikationsmethoden stoßen jedoch in der zunehmend digitalisierten und vernetzten Gesellschaft an ihre Grenzen. Zudem bieten klassische Medien keine oder kaum Möglichkeiten, um die Impfkommunikation den Bedürfnissen der Empfänger*innen anzupassen.

Im vorliegenden Beitrag stellen wir digitale Medien und Technologien für die Impfkommunikation vor, die Vorteile von der personenzentrierten und bedürfnisgerechten Kommunikation zwischen Ärzt*in und Patient*in und von großangelegten und weitestgehend standardisierten Impfkampagnen kombinieren. Wir fokussieren hierbei auf 4 digitale Ansätze: (1) soziale Medien, (2) Smartphone-Apps, (3) Chatbots und (4) immersive Technologien, insbesondere virtuelle Realität.^1^ Wir beschreiben sowohl die Eigenschaften dieser Medien und Technologien als auch mögliche Zielgruppen für ihre Anwendung in der Impfkommunikation. Außerdem fassen wir ausgewählte Evidenz zum Stand der Forschung hinsichtlich der Effektivität dieser digitalen Ansätze in der Impfkommunikation zusammen und identifizieren Forschungslücken und -potenziale. Abschließend diskutieren wir mögliche Anwendungs- und Einsatzbereiche digitaler Innovationen in der Impfkommunikation.

## Soziale Medien

### Eigenschaften und Zielgruppe

Soziale Medien sind digitale Plattformen und Anwendungen, die es Nutz*innen ermöglichen, Inhalte wie Texte, Bilder und Videos zu erstellen, zu teilen und interaktiv miteinander zu kommunizieren. Die Plattformen fördern den Austausch von Informationen und persönlichen Erlebnissen und dienen dem Aufbau und der Pflege von sozialen Netzwerken. Typische Funktionen sozialer Medien umfassen das Liken, Kommentieren und Teilen von Beiträgen sowie das Abonnieren von Kanälen oder Profilen. Zu den bekanntesten sozialen Medien zählen Plattformen wie Facebook, X (ehemals Twitter), Instagram, TikTok, YouTube und LinkedIn, die jeweils auf unterschiedliche Zielgruppen und Inhalte zugeschnitten sind. Soziale Medien spielen eine bedeutende Rolle in der digitalen Kommunikation und beeinflussen zunehmend Meinungsbildung, soziale Interaktionen und den Zugang zu Informationen in der Gesellschaft. Auch wenn soziale Medien zur Verbreitung von Fehlinformationen und zur gesellschaftlichen Polarisierung in Gesundheitsthemen beitragen können, bieten sie gleichermaßen das Potenzial für evidenzbasierte Gesundheitskommunikation, um diesen Tendenzen entgegenzuwirken [10], sowohl durch Einzelpersonen als auch durch Gesundheitsorganisationen und -behörden.

Soziale Medien können das breitenwirksame Element klassischer Medien mit dem interaktiven Element des Beratungsgesprächs vereinen, indem sie es beispielsweise Gesundheitsorganisationen durch öffentliche Kommentarfunktionen ermöglichen, mit der Zielgruppe über die Inhalte einer Impfkampagne in Dialog zu treten (z. B. mit jungen Menschen über die Impfung gegen Gebärmutterhalskrebs), Missverständnissen entgegenzuwirken und Fragen zu beantworten. Die eigenständige Recherche zu Gesundheitsthemen im Internet, einschließlich sozialer Medien, ist weitverbreitet. Laut einer repräsentativen Bevölkerungsstichprobe in Österreich nutzen 64 % der Befragten diese Möglichkeit [11]. Der Zugang zu diesen Informationen über soziale Medien ist dabei niederschwelliger als ein ärztliches Beratungsgespräch.

Wenn Kommunikator*innen gezielt bestimmte Plattformen für eigene Auftritte oder Gesundheitskampagnen auswählen, können verschiedene demografische Gruppen adressiert werden: In der Gruppe der 14- bis 29-Jährigen in Deutschland sind Instagram (79 %) und TikTok (41 %) die meistgenutzten Plattformen; 30- bis 49-Jährige sind überwiegend auf Facebook (50 %) und Instagram (46 %) zu erreichen; bei Personen ab 50 Jahren nimmt die Erreichbarkeit über soziale Medien ab, doch immerhin 28 % der 50- bis 69-Jährigen und noch 14 % der über 70-Jährigen nutzen ebenfalls Facebook [12].

### Evidenzlage: Soziale Medien in der Impfkommunikation

Bisher wurden vor allem 2 Ansätze zur Nutzung sozialer Medien für die Impfkommunikation untersucht. Der erste Ansatz ist der Einsatz bekannter Persönlichkeiten in den sozialen Medien (Influencer), die selbst keine Mediziner*innen sind, die im Rahmen ihrer Inhalte über Impfungen sprechen. Im Vergleich zu klassischen Prominenten ist die Gruppe der Influencer diverser und nahbarer. Das macht es zum Beispiel möglich, sich durch die Auswahl der Influencer mit einer Kampagne an spezifische demografische Gruppen zu wenden [13]. Studien am Beispiel der Influenza-Impfung zeigen, dass Influencer die Einstellungen zur Impfung beeinflussen können. Beispielsweise waren Personen, die Beiträge von Influencern zur Impfung gesehen hatten (verglichen mit einer Kontrollgruppe), eher davon überzeugt, dass die Impfung effektiv ist, man damit sich selbst und andere schützen kann und es sich lohnt, für eine Impfung Zeit und Aufwand zu investieren [13]. Da die Influencer nicht angehalten waren mit den Nutzer*innen zu interagieren (z. B. Kommentare zu beantworten), ist zu vermuten, dass der positive Effekt durch die Veränderung oder Stärkung sozialer Normen zu erklären ist. Darauf deutet auch hin, dass der Großteil der Kommentare unter den Beiträgen positiv war [14], d. h., bereits eher positiv eingestellte Personen wurden ermutigt, sich zu äußern und so die soziale Norm sichtbar zu machen.

Im Gegensatz dazu setzt der zweite Ansatz auf persönliche Interaktion. In einer Studie zur Bereitschaft von Müttern, ihre Kinder impfen zu lassen, befanden sich die Studienteilnehmer*innen in einer WhatsApp-Diskussionsgruppe zum Thema, die von medizinischem Fachpersonal moderiert wurde. Nach Teilnahme an der Diskussionsgruppe empfanden Mütter höhere Selbstwirksamkeit bezüglich der Entscheidung über die Impfung ihrer Kinder, was allerdings nicht in einer höheren Impfrate resultierte [15].

### Forschungslücken und -potenziale

Die Untersuchung der Impfkommunikation von Ärzt*innen über sozialen Medien wie TikTok und Instagram stellt eine Forschungslücke dar. Vielversprechend erscheint die Kombination aus besonderer Glaubwürdigkeit, wenn es sich statt eines nichtmedizinischen Influencers um eine*n Ärzt*in handelt, und einer nahbaren, interaktiven und personalisierten Impfkommunikation. So zeigt eine Studie zur Gesundheitskommunikation durch Ärzt*innen auf TikTok, dass sich Einstellungen gegenüber gynäkologischen Vorsorgeuntersuchungen wie dem PAP-Test durch Kurzvideos von Ärzt*innen merklich verbessern lassen, insbesondere wenn diese in ihrer Kommunikation die Autonomie der Patient*innen betonen und stärken [16].

## Smartphone-Apps

### Eigenschaften und Zielgruppe

Im Bereich der Impfkommunikation werden Smartphone-Apps als digitale Werkzeuge eingesetzt, um Informationen über Impfungen bereitzustellen, das Impfverhalten zu fördern und den Zugang zu Impfangeboten zu erleichtern. Diese Apps können vielfältige Funktionen umfassen, die direkt auf die Bedürfnisse der Nutzer*innen ausgerichtet sind. Smartphone-Apps ermöglichen dabei oft eine direkte und kontinuierliche Kommunikation von Kommunikator*innen mit den Nutzer*innen. Die Apps können jederzeit aktualisiert werden, Benachrichtigungen an Nutzer*innen verschicken und von diesen jederzeit abgerufen werden. Sie ermöglichen zusätzlich einen Grad an Individualisierung, der mit klassischen Medien nicht möglich ist [17]. Eine wichtige Eigenschaft von Smartphone-Apps ist die Möglichkeit von Gamification in der Kommunikation. Gamification bezeichnet die Nutzung spielerischer Aspekte und konnte bereits bei verschiedenen Gesundheitsthemen erfolgreich zur Wissensvermittlung und Verhaltensänderung angewandt werden [18]. Dabei variiert der Einsatz von Gamification von einfachen Quizelementen bis hin zu komplexen Spielen [19].

In Deutschland (wie auch in vielen anderen Ländern) besitzen die meisten Personen ein Smartphone (82 %), wobei die Zahl bei 30- bis 39-Jährigen am höchsten (96 %) und bei Personen über 70 Jahren am niedrigsten (68 %) ist [20]. Vor allem Jugendliche im Alter von 12–19 Jahren nutzen das Smartphone deutlich häufiger als jede andere Form von Medien: Etwa 93 % nutzen ihr Smartphone täglich, während nur 45 % täglich fernsehen, 30 % Radio hören und 11 % Tageszeitungen lesen [21]. Damit bieten Smartphone-Apps die Möglichkeit, einen großen Teil der Bevölkerung in allen Altersgruppen zu erreichen, insbesondere Jugendliche und junge Erwachsene.

### Evidenzlage: Smartphone-Apps in der Impfkommunikation

Insbesondere Smartphone-Apps, deren dargebotener Kommunikationsinhalt an vorher erhobene Einstellungen und Wissensstände der Nutzer*innen angepasst werden kann, erhöhen das Vertrauen in Impfungen. Beispielsweise zeigten Dudley und Kolleg*innen den positiven Einfluss einer App mit individualisierten Inhalten auf das Vertrauen in Impfungen und medizinisches Personal auch noch ein halbes Jahr nach der Intervention [22].

Verschiedene Studien haben zudem den Einfluss von Gamification-Elementen in der Impfkommunikation mit Smartphone-Apps untersucht [23]. In einer Studie wurde Wissen über Mumps, Masern und Röteln und die entsprechende MMR-Impfung mithilfe einer Smartphone-App vermittelt. Dabei bekam eine Gruppe innerhalb der App zusätzlich zur Wissensvermittlung ein Quiz und Punktebelohnungen für die richtige Beantwortung von Fragen. Eltern, die mit der App inklusive Gamification interagierten, hatten nach dem Quiz mehr Wissen und eine höhere Impfintention für ihre Kinder als jene Personen, die die App ohne das Quiz zur Verfügung gestellt bekamen [24]. Ähnliche Ergebnisse zeigen sich auch bei einer Quiz-basierten Smartphone-App mit Studierenden der Gesundheits- und Krankenpflege, die nach Nutzung der App angaben, die Influenza-Impfung eher ihren Patient*innen empfehlen zu wollen [25]. In einer weiteren Studie wurden Eltern und Jugendlichen sowohl Informationen und Impferinnerungen als auch spielerische Lernelemente wie ein Wissensspiel zur HPV-Impfung als Smartphone-App zur Verfügung gestellt [26]. Positive Einstellungen gegenüber der Impfung und das Gefühl, eine informierte Impfentscheidung treffen zu können, waren in der Experimentalgruppe signifikant stärker ausgeprägt als in der Kontrollgruppe, die statt Zugang zur App einen Link zu einer staatlichen Impfinformationsseite erhielt. Zudem ließen sich Jugendliche in der Experimentalgruppe häufiger gegen HPV impfen als in der Kontrollgruppe.

Auch mit komplexeren Spielen können gute Erfolge erzielt werden. Beispielsweise zeigten Ruiz-Lopez und Kolleg*innen, dass ein Rätselspiel, bei dem die Auswirkungen von HPV auf gesunde Zellen sowie die Wirkung von Präventionsmaßnahmen vermittelt wurden, einen deutlichen Wissensanstieg bei den Nutzer*innen erzeugte [27].

### Forschungslücken und -potenziale

Das Potenzial von Smartphone-Apps besteht vor allem darin, personalisierte Inhalte darzubieten und die Auseinandersetzung mit diesen Inhalten über Gamification zu fördern. Neben der Personalisierung der Kommunikationsinhalte anhand des Wissensstandes, der Einstellungen oder demografischer Variablen wäre es vielversprechend, die Kommunikation zukünftig auch hinsichtlich des Ortes und der Zeit zu personalisieren (sogenannte Just-In-Time Adaptive Interventions; [28]). Beispielsweise könnte die Auseinandersetzung mit den Kommunikationsinhalten verstärkt werden, wenn Nutzer*innen Push-Benachrichtigungen erhalten würden, während sie zu Hause und nicht am Arbeitsplatz sind. Die Einladung zur Beantwortung eines Impfquiz könnte kurz vor einem geplanten Arzttermin erfolgen, um darin aufkommende Fragen mit dem/der Ärzt*in zu besprechen. Weiterhin sollte untersucht werden, ob die Effektivität von solchen Smartphone-Apps erhöht werden kann, wenn nicht nur die Kommunikationsinhalte, sondern auch die Gamification-Elemente an die Vorlieben der Nutzer*innen angepasst werden.

## Chatbots

### Eigenschaften und Zielgruppe

Chatbots sind interaktive Programme oder Online-Anwendungen, die in Echtzeit Konversationen mit Nutzer*innen simulieren. Sie werden schon lange in der Gesundheitskommunikation genutzt [29, 30]. Vor den großen Fortschritten insbesondere im Bereich der generativen künstlichen Intelligenz (KI) in den vergangenen Jahren waren Chatbots häufig statisch bzw. regelbasiert. Das bedeutet, dass sie spezifische Fragen anhand vorgegebener Antworten aus einer Datenbank beantworteten. Häufig mussten Nutzer*innen eine Frage aus einer vorgegebenen Auswahl von Optionen auswählen und erhielten dann darauf eine Antwort. Die Eingabe eigener Fragen war kaum möglich. Durch die Anwendung von generativer KI, insbesondere sogenannter Large Language Models (LLM), können Chatbots inzwischen überzeugend menschliche Sprache interpretieren und ausgeben, sowohl in text- als auch in stimmbasierter Form [29], was Nutzer*innen zu weitestgehend natürlichen Interaktionen einlädt. Diese Chatbots werden mit spezifischen Fachinhalten trainiert, um eine korrekte Wissensbasis als Grundlage für die Antworten zu ermöglichen. Diese neue Generation von Chatbots kann auch über die Trainingsinhalte hinaus hilfreiche Antworten liefern und andere Quellen oder Anlaufstellen nutzen, um relevante Informationen bereitzustellen.

In der Gesundheitskommunikation können Chatbots konkrete Fragen von Nutzer*innen beantworten, aber auch empathisch auf deren Sorgen eingehen [31, 32]. Die Anonymität in der Anwendung erleichtert auch den Austausch ohne Angst vor Stigmatisierung aufgrund bestimmter Fragen oder Sorgen [33]. Es gibt dabei auch keine Wartezeiten oder zeitliche Begrenzungen, wie sie bei Beratungsgesprächen mit Ärzt*innen auftreten können, sodass Chatbots auch zur Reduzierung der Belastung für medizinisches Personal beitragen können. Chatbots können über verschiedene Plattformen bereitgestellt werden, zum Beispiel über Webbrowser oder Messenger-Apps, wie WhatsApp, soziale Medien oder in eigens dafür konzipierten Desktop- oder Smartphone-Apps [32]. Damit können sie einem Großteil der Bevölkerung zugänglich gemacht werden.

### Evidenzlage: Chatbots in der Impfkommunikation

Obwohl Chatbots bereits seit einiger Zeit in der Gesundheitskommunikation allgemein und spezifisch in der Impfkommunikation eingesetzt werden, ist die Evidenz zur Effektivität sehr gemischt. In Studien zur Impfkommunikation wird häufig die Impfintention als wichtiger Prädiktor für tatsächliches Verhalten untersucht. Eine Metaanalyse über 5 randomisierte kontrollierte Studien mit Chatbot-Interventionen fand keinen signifikanten Effekt auf die Impfintention [32]. Von den untersuchten Chatbots nutzte allerdings nur einer generative KI. In dieser Studie wurden Eltern in einer Zeitspanne von 12 Wochen regelmäßig an die bevorstehende Impfung ihrer Kinder erinnert. Die Interventionsgruppe erhielt zusätzlich Informationen und motivierende Nachrichten durch einen Chatbot via Messenger-App. Eltern in dieser Interventionsgruppe hatten eine höhere Impfintention, eine gesteigerte Motivation und Selbstwirksamkeit als Personen in der Kontrollgruppe, die nur eine Broschüre zur Information erhielt [34].

Darüber hinaus konnten bereits einige Studien positive Effekte von Chatbots auf das Wissen und die Einstellung zum Impfen nachweisen. In einem systematischen Review zur Chatbot-Nutzung in der Impfkommunikation zeigten alle Studien positive Effekte bezüglich des Impfwissens und der Einstellung [29], ohne jedoch Langzeiteffekte zu betrachten. Auch die Metaanalyse von Chan et al. [32] konnte durch Chatbot-Interventionen einen positiven Effekt auf die Einstellung gegenüber Impfungen finden, sowohl allgemein als auch für spezifische Impfstoffe, für die eigene Impfung und die Impfung anderer (Kinder und ältere Menschen). In qualitativen Interviews zum Einsatz eines KI-Chatbots als digitaler Assistent zur Beratung bei der Impfung gegen Covid-19 in Hongkong konnten neben einem verbesserten Impfwissen auch eine erhöhte Gesundheitskompetenz und ein höheres Vertrauen in Impfungen bei den Nutzer*innen gezeigt werden [35].

Um den möglichen Nutzen von Chatbots für die Gesundheitskommunikation zu ermitteln, ist es zudem wichtig, deren Nutzer*innenfreundlichkeit zu betrachten. Laut einem systematischen Review werden Interaktionen mit Chatbots als natürlicher wahrgenommen, wenn es interaktive Funktionen gibt, Informationen personalisiert werden, Antworten kurz und klar sind und Bilder oder Videos integriert werden [29]. Lange und sich wiederholende Texte, offensichtliche Wissenslücken und ein roboterhaftes Gefühl im Umgang führen zu einem schlechteren Erleben bei den Nutzer*innen. Die Autor*innen betonen, dass es wichtig sei, vor der Nutzung zu kommunizieren, was ein Chatbot kann und was nicht, und bei Lücken auf weitere Quellen und Anlaufstellen hinzuweisen. Während der Covid-19-Pandemie wurde von der Weltgesundheitsorganisation (WHO) ein Chatbot namens Florence vorgestellt, um über die Pandemie und die Impfung gegen Covid-19 aufzuklären. In einer Studie wurden den Teilnehmer*innen die wichtigsten Eigenschaften des Chatbots vor der Nutzung kommuniziert, was bei ihnen zu einer höheren Zufriedenheit mit dem Chatbot führte [36]. Die Autor*innen fanden zudem positive Effekte auf die Nutzer*innenzufriedenheit durch die erlebte Neuheit und den Trend der Technologie sowie durch Kontrollerleben, Interaktivität und Einfachheit in der Nutzung des Chatbots bei der Suche nach Informationen. Einen negativen Einfluss auf die Nutzer*innenzufriedenheit hatte die Sorge um die eigene Privatsphäre und Datensicherheit. Die Studie zeigte außerdem, dass eine höhere Zufriedenheit mit dem Chatbot sich auch positiv auf die Beziehung zur Gesundheitsorganisation auswirkte: Zufriedenere Nutzer*innen interagierten vermehrt öffentlich mit der Online-Präsenz der WHO, z. B. durch das Teilen ihrer Inhalte in sozialen Medien.

### Forschungslücken und -potenziale

Die bisherigen Studien zeigen eine große Heterogenität in der Art der eingesetzten Chatbots. Reviews und Metaanalysen greifen neben KI-gestützten Chatbots vor allem noch ältere Technologien mit statischen bzw. regelbasierten Chatbots auf [29, 32]. Aktuelle Studien mit KI-gestützten Chatbots zeigen vielversprechende Ergebnisse in der Impfkommunikation. Insbesondere Aspekte der Nutzerfreundlichkeit werden durch KI gestützte Chatbots besser adressiert: Konversationen erscheinen natürlich und fließend und es können sogar gesprochene Eingaben verarbeitet und beantwortet werden. Um die Effekte KI-gestützter Chatbots zu bewerten und mögliche Risiken zu verstehen (z. B. das Vermitteln falscher Informationen) bedarf es jedoch noch substanzieller Evidenz.

KI-gestützte Chatbots können anhand von Eingaben der Nutzer*innen Stimmungen und Einstellungen erkennen und personalisiert antworten [37]. Besonders bei impfskeptischen Personen kann eine empathische Kommunikation durch den Chatbot effektiver sein [38]. Dies bietet großes Potenzial und erfordert weitere empirische Überprüfungen.

In zukünftigen Evaluationsstudien sollte die Effektivität von Chatbots mit klassischen Informationsmedien verglichen werden. Derartige kontrollierte Interventionsstudien gibt es bisher kaum, sie könnten aber wichtige Informationen über die Möglichkeiten des Einsatzes von Chatbots als komplementäres Medium in der Impfkommunikation liefern.

## Immersive Technologien

### Eigenschaften und Zielgruppe

Immersive Technologien zielen darauf ab, die Nutzer*innen in eine virtuelle oder künstliche Umgebung zu versetzen, sodass diese das Gefühl haben, physisch in dieser Umgebung präsent zu sein. Immersive Technologien variieren darin, wie stark die reale Welt in die künstliche Umgebung integriert wird: Während „erweiterte Realität“ (Augmented Reality, AR) Elemente der echten und virtuellen Realität kombiniert, exkludiert „virtuelle Realität“ (Virtual Reality, VR) die reale Welt nahezu komplett und schafft eine vollständig virtuelle Umwelt. Das immersive Erlebnis ist dadurch gekennzeichnet, dass sich Nutzer*innen in der virtuellen Umgebung anwesend fühlen, sie das Gefühl haben, dass der virtuelle Körper tatsächlich der eigene Körper ist und Kontrolle über Handlungen und deren Ergebnisse zu erleben. Diese Eigenschaften können immersive Technologien (insbesondere VR) zu einer exzellenten Methode erfahrungsbasierten Lernens in der Gesundheitskommunikation machen [39]. In der Impfkommunikation können damit Dinge erlebbar gemacht werden, die in der Realität nicht direkt oder nur zeitverzögert erlebt werden (z. B. die Übertragung von Virusinfektionen und der indirekte Schutz durch Herdenimmunität, auch aus Perspektive anderer Personen).

Etwa die Hälfte der Besitzer*innen von VR-Brillen sind in der Altersgruppe zwischen 18 und 34 Jahren [40], allerdings wächst das Interesse an diesen Technologien und die Heterogenität der Nutzer*innen international stetig, auch in Deutschland [41]. Dennoch sind immersive Technologien in der Impfkommunikation aktuell wohl vor allem für junge Erwachsene interessant, insbesondere weil der Konsum klassischer Medien bei dieser Gruppe stark rückläufig ist [21] und damit alternative Kommunikationsmedien besondere Relevanz haben.

### Evidenzlage: Immersive Technologien in der Impfkommunikation

Mehrere Studien konnten unter der Verwendung unterschiedlicher VR-Umwelten und -Simulationen zeigen, dass die Bereitschaft, sich selbst und vulnerable Personen zu schützen, und damit auch die Impfbereitschaft stieg, wenn die Effekte der Herdenimmunität in VR erlebt werden konnten [42–46]. Die Interventionseffekte überstiegen dabei die Effektivität anderer Kommunikationsmethoden (z. B. Text, Video; [44, 46, 47]) und waren auch noch mehrere Tage nach der Intervention stabil [43]. Zudem gibt es erste Hinweise darauf, dass die Interventionseffekte am besten durch erfahrungsbasiertes Lernen erklärt werden können, aber nicht durch die Neuartigkeit der Technologie (und eine damit verbundene positivere Einstellung zum Kommunikationsthema) oder durch gesteigerte Empathie für andere Personen [45]. Erste Studien zeigen zudem, dass auch AR-Simulationen die Impfbereitschaft erhöhen können, wenn auch weniger effektiv als VR [48].

### Forschungslücken und -potenziale

Die aktuellen VR-Studien beschränken sich auf Impfkommunikation zu Covid-19 und Influenza; zukünftige Forschung muss die Generalisierbarkeit für andere Impfungen zeigen. Zudem verwendeten diese Studien VR-Simulationen, die nicht auf die Bedürfnisse oder verschiedene Vorerfahrungen der Nutzer*innen angepasst waren. Unter Verwendung von generativer KI könnten solche Simulationen zukünftig adaptiv(er) und damit wahrscheinlich auch effektiver werden (z. B. indem Nutzer*innen echte Dialoge mit nichtmenschlichen VR-Agent*innen führen können). Weiterhin steht die Forschung zu den zugrunde liegenden Prozessen, die die Effektivität von VR-Interventionen erklären und mit anderen Interventionsmethoden vergleichen, noch am Anfang (z. B. [47]). Wenn richtig verstanden wird, *warum* immersive Technologien so gut funktionieren, kann die Technologie noch effektiver für die Impfkommunikation einsetzt werden. Bisherige theoretische und empirische Arbeiten legen nahe, dass insbesondere die erfahrungsbasierte Kommunikation (vs. informationsbasierte Kommunikation) zu besonders starken Interventionseffekten führen kann [39], allerdings ist hierzu noch weitere Evidenz zu den zugrunde liegenden Effekten erforderlich.

## Anwendungs- und Einsatzmöglichkeiten digitaler Innovationen in der Impfkommunikation

Soziale Medien sind beliebt, wobei die Nutzung verschiedener Plattformen je nach Altersgruppe variiert. Gesundheitsorganisationen und Ärzt*innen könnten diese Plattformen nutzen, um über Impfungen zu informieren und insbesondere auch um Fehlinformationen sowie Verschwörungserzählungen entgegenzuwirken, die dort verbreitet werden [49].

Die potenzielle Reichweite für Impfkommunikation über Smartphone-Apps ist noch größer; allerdings müssen die Nutzer*innen diese Apps erst auf ihrem Smartphone installieren. Hier könnte eine umfassende Gesundheits-App zielführend sein, die verschiedene Angebote und Informationen bündelt. Ein Vorteil dieser Technologie ist die mögliche Integration von Buchungs- und Erinnerungssystemen. Gamifizierte Wissensspiele könnten außerdem im Bildungsbereich (z. B. in Schulen) eingesetzt werden. Hier ist jedoch der Nachteil gegenüber sozialen Medien, dass diese Apps bewusst geöffnet und genutzt werden müssen, während Informationen, die über soziale Medien verbreitete werden, auch ohne das bewusste Aufsuchen rezipiert werden können.

Chatbots werden zunehmend auf Webseiten von Behörden und Gesundheitsorganisationen genutzt. Ärzt*innen sollten auf diese Angebote verweisen und Gesundheitsbehörden könnten es erleichtern, zertifizierte Chatbots auch auf Praxis-Webseiten zu integrieren. Dies würde ein komplementäres Beratungsangebot schaffen und Ärzt*innen entlasten.

Die Verfügbarkeit der Hardware (z. B. VR-Brillen) ist die größte Hürde für den breiten Einsatz immersiver Technologien in der Impfkommunikation. Dennoch könnten diese bereits in Arztpraxen (z. B. im Warteraum) und im Bildungsbereich zum Einsatz kommen.

Für alle hier aufgeführten digitalen Ansätze in der Impfkommunikation gilt die Einschränkung, dass sie eine gewisse Motivation der Zielpersonen erfordern, sich mit dem Thema Impfen auseinanderzusetzen. Wenn die Zielperson das Thema grundsätzlich vermeidet oder eine starke negative Einstellung zum Impfen hat, dann werden digitale Ansätze in der Impfkommunikation ebenso wenig erfolgreich sein wie andere Kommunikationsansätze. Eine Einschränkung der aktuellen Forschung zur Anwendbarkeit ist außerdem, dass es bisher keine systematischen Untersuchungen dazu gibt, welche Kommunikationsinhalte am besten mit welcher Kommunikationsmethode vermittelt werden können, auch wenn die meisten der vorgestellten digitalen Ansätze neutral gegenüber dem Kommunikationsinhalt sind.

## Fazit

In einer zunehmend digitalisierten Welt eröffnen soziale Medien, Smartphone-Apps, Chatbots und immersive Technologien innovative Möglichkeiten, um breite Bevölkerungsgruppen effizienter zu erreichen, Informationen gezielt zu vermitteln und Fehlinformationen entgegenzuwirken oder positive Effekte von Impfungen erlebbar zu machen (für einen Überblick siehe Tab. 1). Die Integration solcher digitalen Ansätze in bestehende Kommunikationsstrategien kann einen entscheidenden Beitrag zur Stärkung zukünftiger Impfkampagnen und zur Förderung der globalen Gesundheit leisten.

**Tab. 1:** Übersicht digitaler Innovationen in der Impfkommunikation

Technologie	Zielgruppe	Anwendungsbereich	Ausgewählte Forschungspotenziale
Soziale Medien	Jugendliche und Erwachsene aller Altersgruppen, wobei Plattformnutzung und Nutzungsintensität zwischen Altersgruppen variiert	Nichtmedizinische Influencer, medizinisches Fachpersonal oder Gesundheitsorganisationen und Behörden auf sozialen Medien	Effekte unterschiedlicher Kommunikator*innen (z. B. medizinische Laien vs. Expert*innen)
Smartphone-Apps	Jugendliche und Erwachsene aller Altersgruppen	Personalisierte und gamifizierte Apps in Kombination mit Buchungs- und Erinnerungssystemen über Gesundheitsorganisationen und Behörden	Effekte der Personalisierung unterschiedlicher Kommunikationsinhalte und -formen
Chatbots	Jugendliche und Erwachsene aller Altersgruppen	Evidenzbasierte und zertifizierte Chatbots auf Webseiten von Gesundheitsorganisationen, Behörden und Praxen von Ärzt*innen	Potenziale von generativer KI bei Chatbots
Immersive Technologien	Insbesondere Jugendliche und junge Erwachsene	Immersive Apps (z. B. VR-Apps) in Praxen von Ärzt*innen oder im Bildungsbereich; bei zunehmender Verbreitung der Hardware in der Bevölkerung auch über entsprechende App-Plattformen	Potenziale von immersiven Apps, die z. B. mithilfe von generativer KI personalisiert werden

*KI* künstliche Intelligenz,* VR* Virtual Reality

## Footnotes

1 In diesem Beitrag beschränken wir uns auf digitale Ansätze, die Informationen zur Krankheit oder dem Nutzen von Impfen kommunizieren, während digitale Einladungs- und Erinnerungssysteme in einem anderen Beitrag dieses Themenhefts vorgestellt werden (siehe Beitrag von Takla und Wulkotte et al.).
